# A Case of Alveolar-Cell Carcinoma Misdiagnosed As Tubercular Bronchopneumonia

**DOI:** 10.7759/cureus.31371

**Published:** 2022-11-11

**Authors:** Ulhas Jadhav, Gaurang M Aurangabadkar, Puja Upadhyay, Mrinmayee V Mayekar, Saran K Chacko, Srinivasulareddy Annareddy

**Affiliations:** 1 Respiratory Medicine, Jawaharlal Nehru Medical College, Datta Meghe Institute of Medical Sciences, Wardha, IND; 2 Respiratory Medicine, Datta Meghe Medical College, Datta Meghe Institute of Medical Sciences,, Nagpur, IND; 3 Respiratory Medicine, Jawaharlal Nehru Medical College, Datta Meghe Institue Of Medical Sciences, Wardha, IND

**Keywords:** ct guided biopsy, acid-fast bacilli, bronchopneumonia, tuberculosis, bronchoalveolar cell carcinoma

## Abstract

Tuberculosis (TB) and cancer are two of the most prevalent disease across the globe. Cases of lung cancer are increasing rapidly and have now reached almost epidemic levels throughout the world. The two diseases share various radiological features and symptoms and coming to a diagnosis sometimes becomes challenging. In a situation like this, an invasive procedure to establish a diagnosis becomes necessary. We report a case of 35-year-old female presenting with cough and dyspnea, initially diagnosed as pulmonary bronchopneumonia and later found to have alveolar-cell carcinoma.

## Introduction

Tuberculosis (TB) and malignancies are two of the most common illnesses that affect humans on a global scale. TB remains a major cause of illness and death. Each year, 8.8 million additional new cases of TB are reported worldwide [1]. Prior to the turn of the century, lung cancer in India was thought to be rare. However, since the last decade, it is slowly becoming more common. According to estimated figures, it affects 1.61 million people annually and is the primary cause of cancer-related death among men globally [1]. Due to the varying clinical presentations and a paucity of suitable diagnostic testing facilities, TB has frequently been diagnosed mistakenly in cancer patients [2]. We report the case of a 35-year-old female patient, who was conclusively diagnosed as a case of alveolar-cell carcinoma, after an initial misdiagnosis as tubercular bronchopneumonia. This case highlights the confusing and varied clinical and radiological presentation of bronchoalveolar-cell carcinoma (BAC), which can mimic bronchopneumonia.

## Case presentation

A 35-year-old female patient came to the casualty department with chief complaints of progressively increasing dyspnea on exertion for the previous four months that steadily worsened in severity. The patient also reported diffuse, dull, painful chest pain for the last three months, along with a cough with mucoid expectoration. The patient was put on antitubercular medication based on clinical and radiological results at an outpatient hospital, where she was primarily diagnosed as having pulmonary TB. Detailed clinical examination findings in the patient are summarized in Table 1.

**Table 1:** Summary of clinical examination findings of the patient

Clinical examination parameters	Clinical findings of the patient
Pulse rate	70 beats per minute
Respiratory rate	30 breaths per minute
Blood pressure	130/90 mmHg
Oxygen saturation on room air	85% on room air
Lymph node examination	A single lymph node measuring 2 x 1 cm palpable in the right submandibular area
Chest auscultation	Bilateral crepitations present over all lung fields

**Figure 1:**
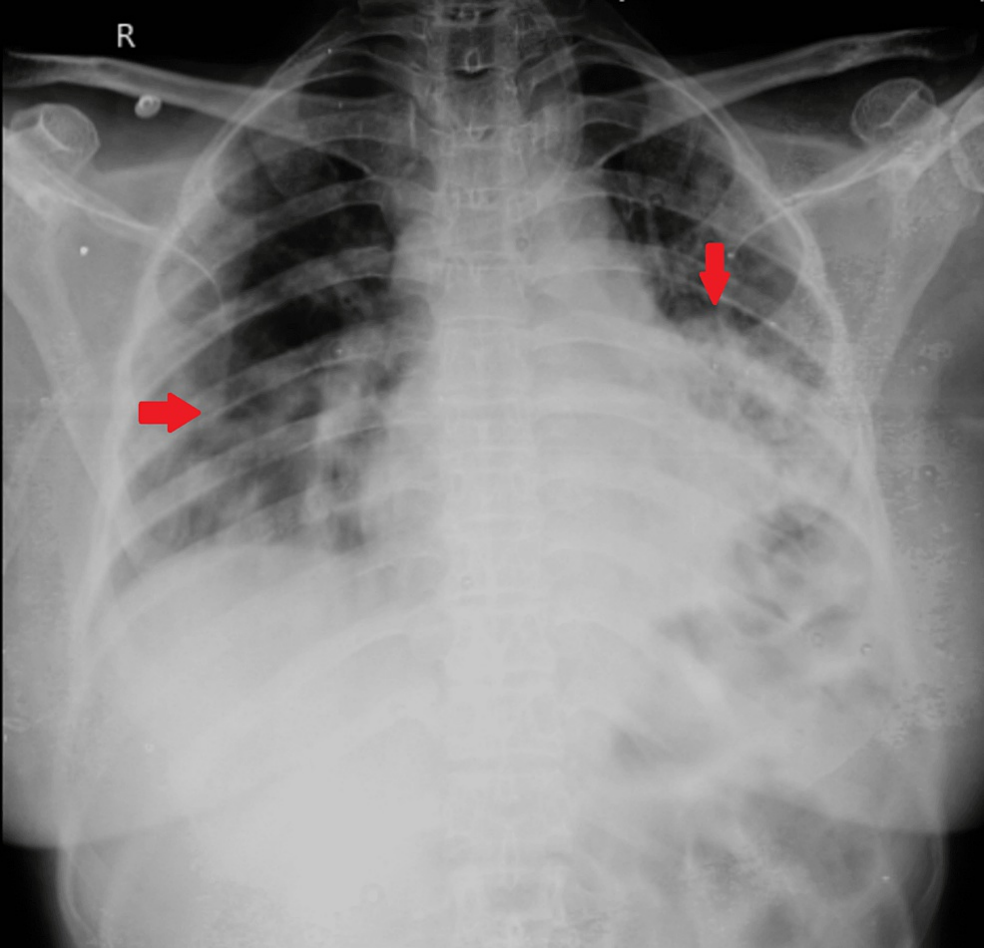
A chest X-ray posteroanterior (PA) view of the patient showing fluffy opacities in the right lower, left middle, and lower zone (red arrows)

Routine blood investigations were done, which were unremarkable. Arterial blood gas (ABG) analysis was done, which was suggestive of hypoxia. A chest X-ray posteroanterior (PA) view was done, which showed fluffy opacities in the right lower, left middle, and lower zones (Figure 1).

**Figure 2:**
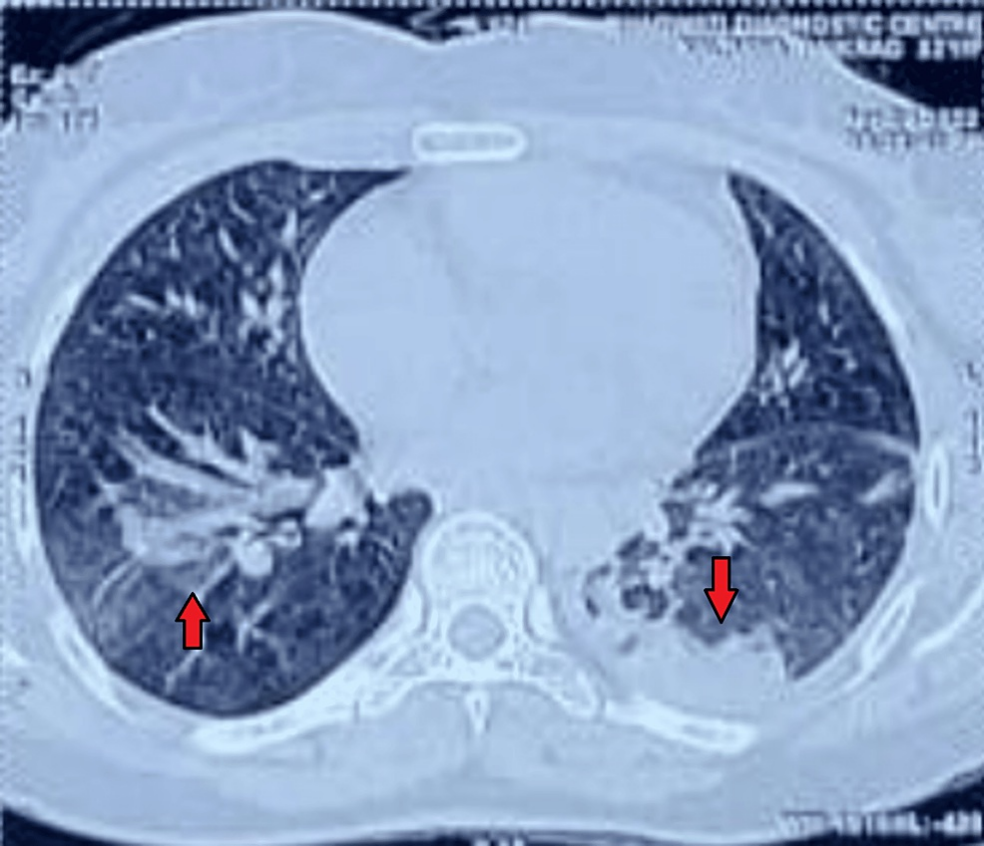
High-resolution computerized tomography (HRCT) scan of the chest showing well-defined, solid, contrast-enhancing nodules in bilateral lung fields and a left lower lobar consolidation (red arrows)

A provisional diagnosis of tubercular bronchopneumonia was made based on the clinical history and radiological findings of the patient and was started on anti-tubercular therapy. However, sputum samples that were sent for acid-fast bacilli (AFB) staining were found to be negative. A diagnostic fiber-optic bronchoscopy was undertaken and no obvious airway abnormality or endobronchial lesions were found. Bronchoalveolar lavage (BAL) samples were negative for AFB and cytology did not reveal any evidence of malignant cells. The absence of any clinical improvement in the patient’s condition warranted further imaging investigations and, therefore, a high-resolution computerized tomography (HRCT) scan of the chest was done, which was suggestive of well-defined, solid, contrast-enhancing nodules in bilateral lung fields and a left lower lobar consolidation (Figure 2).

**Figure 3:**
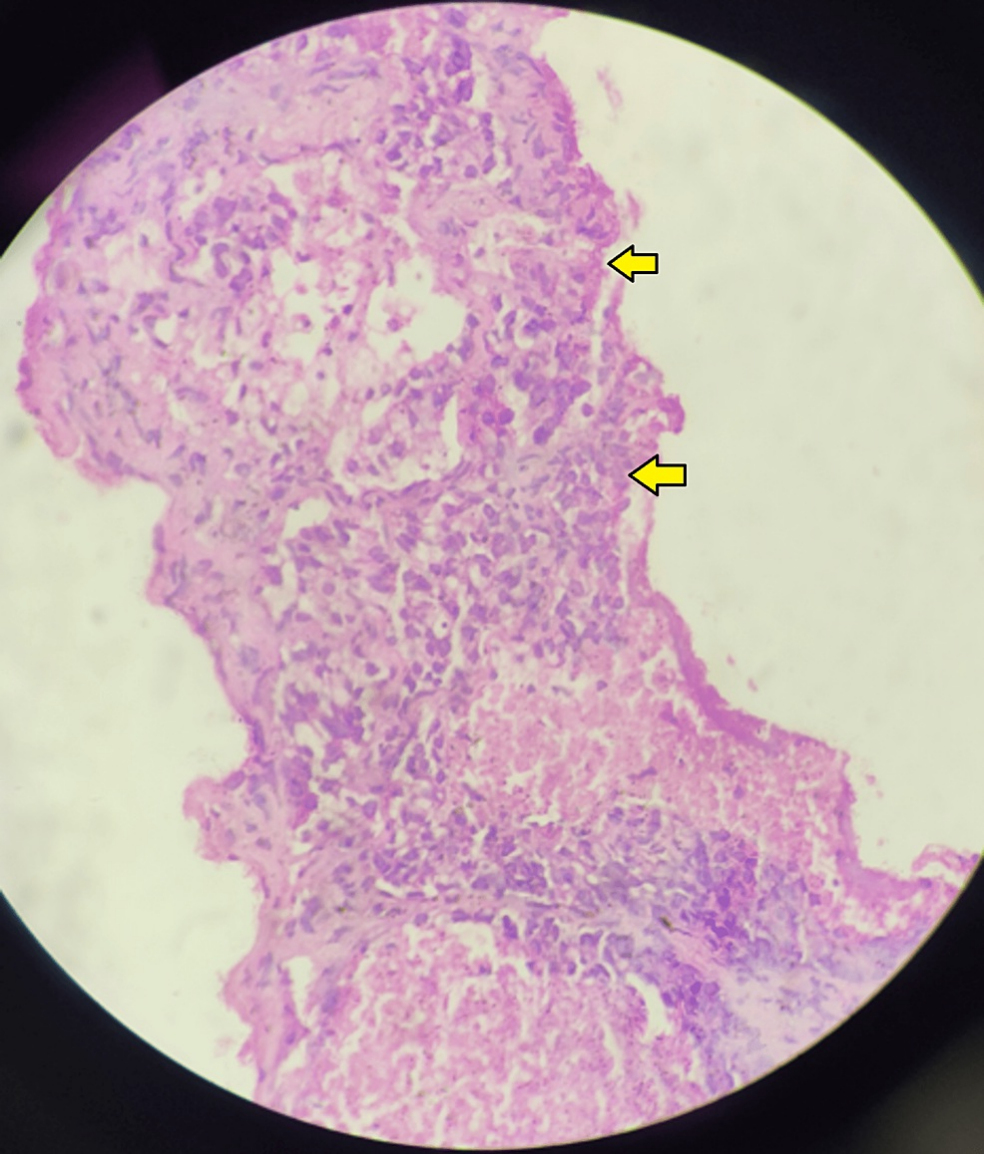
Histopathological examination (HPE) of the computerized tomography (CT)-guided lung biopsy showing fibro-collagenous stroma along with scattered sheets of severely atypical epithelial cells of glandular origin, suggestive of bronchoalveolar-cell carcinoma (BAC) (yellow arrows)

The presence of solid enhancing nodules on contrast study raised our index of suspicion for a possible malignant etiology and, therefore, a trans-thoracic CT guided biopsy was done and a tissue sample was sent for histopathological examination (HPE). The tissue section on HPE showed fibro-collagenous stroma along with scattered sheets of severely atypical epithelial cells of glandular origin suggestive of BAC, which clinched our diagnosis (Figure 3).

A medical oncologist consultation was taken once the diagnosis was made, and the patient was started on a chemotherapy regimen with carboplatin and paclitaxel, to which the patient responded well, and demonstrated substantial clinical improvement. She was advised to review for the next cycle of chemotherapy after three weeks.

## Discussion

Lung cancer is the cause of 5.9% of all cancer cases and 8.1% of all cancer-related deaths in India [3]. Adenocarcinoma with a lepidic pattern, formerly known as BAC, is a diverse group of tumors that have lepidic development dispersed along alveolar septa without stromal, pleural, or vascular invasion as a common pathologic feature, and is a type of non-small cell carcinoma [4].

In a country with a high-TB burden such as India, radiological findings and clinical findings can lead to a differential diagnosis of TB instead of malignancy as highlighted in our case report. The WHO TB statistics for India for 2021 give an estimated incidence figure of 2.59 million active TB cases. This translates to a rate of 188 TB cases per 100,000 people [5]. Because of its high prevalence and radiological similarities, lung cancer patients are often misdiagnosed as pulmonary TB based on radiological pictures alone. In a study to determine what delays the diagnosis of lung cancer, it was discovered that out of 123 patients with lung cancer, 23 patients (17%) had been initially wrongly diagnosed with pulmonary TB [6]. Unproductive and unwarranted anti-TB treatment greatly lengthens this delay [6].

## Conclusions

In a TB-endemic nation such as India, where pulmonary TB can present with any radiological appearance and can mimic symptoms of any pulmonary disease, this case report emphasizes the necessity of maintaining a high index of suspicion in patients who are non-responders to anti-tubercular therapy and showing progressive radiological worsening. Taking extra efforts to do further invasive investigations such as imaging-guided biopsies, in order to reach a diagnosis can be richly rewarding for the clinician and of utmost benefit to the patient. Therefore, all patients of non-resolving pneumonia who show no radiological improvement even after broad-spectrum antimicrobial therapy should undergo an imaging-guided lung biopsy to reach towards a suitable diagnosis.
